# Isolation of Rat and Human Hippocampal Neuron Fractions in a Discontinuous Density Gradient

**DOI:** 10.1100/tsw.2002.850

**Published:** 2002-06-14

**Authors:** John Graham

**Affiliations:** School of Biomolecular Sciences, Liverpool John Moores University, UK

**Keywords:** neurons, rat hippocampus, human hippocampus, neuronal culture, OptiPrep^TM^, iodixanol, discontinuous density gradient

## Abstract

The plating efficiency of neurons in culture is highly dependent on the concentration of cells used to establish the monolayer. A discontinuous iodixanol gradient permits both the production of a viable concentrated suspension of neurons and purification from other brain tissue elements. The gradient that is described in this Protocol Article is applicable to brain tissue from rat and also from human biopsy specimens.

**METHOD TYPE:** extraction, isolation, purification and separation

**SUB METHOD TYPE:** centrifugation

## INTRODUCTION

Marks et al.[1] observed that the plating out of neurons directly from rat hippocampi after enzymic disaggregation and mechanical triturition became notably less efficient as the age of the animals increased from 8 to 35 days. However poor plating efficiency could be overcome by increasing the cell concentration in the suspension applied to substratum. This was achieved by banding the neurons in a discontinuous iodixanol gradient. The four-step gradient covered the density range 1.026–1.055 g/ml. A similar strategy was used by Liu et al.[2] although the density range was slightly different and Brewer et al.[3] extended the OptiPrep™-based methodology to human cortical tissue obtained at surgery; the density range of the gradient was 1.031–1.061 g/ml.

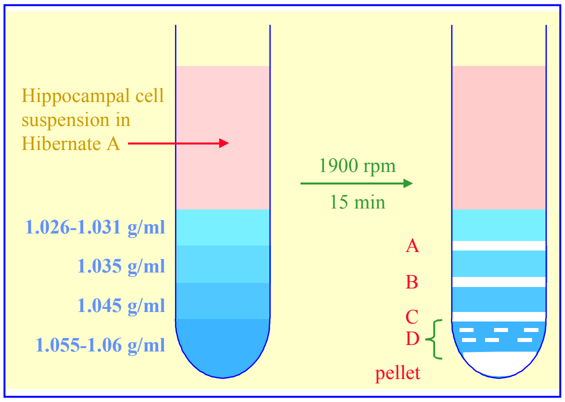

**FIGURE 1**. Purification of hippocampal neurons (see text for more information).

In all cases material is observed at each interface and in a pellet. Although neurons are present throughout the gradient, they are most highly enriched in the densest layer between the lowest interface and the pellet (see Fig. 1). Sometimes only this layer is harvested[3], sometimes the pellet is mixed with the lower layer after discarding the upper layers[1].

Techniques for disaggregating the tissue using enzymic digestion and trituritionare not included in this protocol. They are complex; vary significantly from laboratory to laboratory and outside the author's area of expertise. One method is described in detail in Ref. [4]. This protocol (adapted from Ref. [3]) confines itself therefore to a description of the preparation of gradient solutions; the gradient centrifugation step and some of the variations described in the Notes.

## MATERIALS AND EQUIPMENT

OptiPrep™ (60%, w/v iodixanol)
OptiPrep™ Diluent: see Note 1
Working Solution (WS) of 30%, w/v) iodixanol: mix equal volumes of OptiPrep™ and OptiPrep™ Diluent
Suspension Medium (SM): see Notes 2 and 3

Plastic conical centrifuge tubes (12–15 ml)
Syringe with metal cannula (for underlayering) and/or plastic Pasteur pipette (for overlayering)
Low-speed (temperature-controlled) centrifuge with swinging-bucket rotor

## METHOD

1. Prepare a single cell suspension from the tissue by enyzmic disaggregation and trituition[1,2,3,4].
2. Suspend the cells in the SM of choice, this is often Hibernate A (but see Notes 2 and 3), which can be supplemented as required[1,2,3].
3. Prepare density gradient solutions of the following density (1.06, 1.045, 1.035, and 1.03 g/ml) by dilution of 1 ml of WS with 1.9, 3.0, 4.4, and 5.5 ml, respectively, of SM (see Notes 4 and 5).
4. In a 15-ml centrifuge tube layer 1 ml of each of the four density solutions and then 6 ml of the cell suspension on top (see Note 6).

5. Centrifuge at 1,900 rpm for 15 min at room temperature. Turn the brake off during the deceleration.
6. The distribution of material in the gradient is shown in Fig. 1. Brewer et al.[3] collected the fraction marked D, while Marks et al.[1] included the pelleted material in the neuron harvest (see Note 7).
7. Dilute the fraction with 1–2 vol of SM and harvest the cells by centrifugation.

## NOTES

1. The choice of OptiPrep™ diluent may vary with operational requirements. Brewer et al.[3] observed that saline (0.8–0.9% NaCl) buffered with 10 mM MOPS-NaOH, pH 7.4 gave superior results. This diluent may however be any buffered isoosmotic solution; it may be, for example, Hibernate A (see Note 3). Refs. [5] and [6] give more information about gradient solution preparation.
2. The SM may be Hibernate A (see Note 3) or any suitable medium that is compatible with neurons. Liu et al.[2] for example used a serum-free growth medium based on DMEM.
3. For sources of Hibernate A contact Dr. Gregory Brewer, Dept. of Molecular Biology, Microbiology and Biochemistry, South Illinois University School of Medicine, Springfield, IL 62794-9626 (gbrewer@siumed.edu).
4. The optimal density of the four layers may require some experimentation. For neurons from other species in particular, it may be necessary to modulate the density of the layers. Precise densities should be adjusted in the light of experience. More information on preparation of solutions of different densities can be obtained from Ref. [5]. The gradient used by Marks et al.[2] for example could comprise equal volumes of 1.026, 1.035, 1.045, and 1.055 g/ml, approx. equivalent to 3.8, 5.4, 7.3, and 9.2% (w/v) iodixanol. The centrifugation conditions were the same — 1,900 rpm for 15 min.
5. The inclusion of a denser layer might be considered (e.g., 1.07 g/ml) if some denser contaminants are present. Whether improved resolution could be obtained by loading the sample in a dense solution below the gradient is another operational variant that might be considered.
6. Information on the construction of discontinuous density gradients can be found in Ref. [6].
7. The optimal harvesting procedure should be worked out once the composition of the zones of banded material (A–D in Fig. 1) and the pellet have been verified.

## ACKNOWLEDGEMENTS

The author and TheScientificWorld wish to thank Axis-Shield PoC, AS, Oslo, Norway for their kind permission to adapt OptiPrep™ Application Sheet C22 in the preparation of this Protocol Article and Dr. Gregory Brewer, Dept. of Molecular Biology, Microbiology and Biochemistry, South Illinois University School of Medicine, Springfield, IL 62794-9626, for his helpful comments in the preparation of the protocol.
